# Perinatal depression screening practices in a large health system: identifying current state and assessing opportunities to provide more equitable care

**DOI:** 10.1007/s00737-020-01035-x

**Published:** 2020-05-05

**Authors:** Abbey Sidebottom, Marc Vacquier, Elizabeth LaRusso, Darin Erickson, Rachel Hardeman

**Affiliations:** 1 Care Delivery Research, Allina Health, 800 E 28th Street, MR 15521, Minneapolis, MN USA; 2 Mental Health Clinic, Allina Health, 800 E 28th Street, Minneapolis, MN USA; 3 Division of Epidemiology and Community Health, School of Public Health, University of Minnesota, 1300 S 2nd St, Minneapolis, MN USA; 4 Division of Health Policy and Management, School of Public Health, University of Minnesota, 420 Delaware St SE, Minneapolis, MN USA

**Keywords:** Prenatal depression, Postpartum depression, Screening, Disparities

## Abstract

The purpose of this study was to assess the prevalence of prenatal and postpartum depression screening in a large health system and to identify covariates for screening, with a specific focus in understanding disparities in practice. A retrospective cohort of women with deliveries in 2016 was created using electronic health records. Primary outcomes were depression screening during pregnancy and the first 3 months postpartum. Generalized linear mixed models with women nested within clinic were used to determine the effect of maternal and clinical characteristics on depression screening. The sample included 7548 women who received prenatal care at 35 clinics and delivered at 10 hospitals. The postpartum sample included 7059 women who returned within 3 months for a postpartum visit. Of those, 65.1% were screened for depression during pregnancy, and 64.4% were screened postpartum. Clinic site was the strongest predictor of screening, accounting for 23–30% of the variability in screening prevalence. There were no disparities identified with regard to prenatal screening. However, several disparities were identified for postpartum screening. After adjusting for clinic, women who were African American, Asian, and otherwise non-white (Native American, multi-racial) were less likely to be screened postpartum than white women (AOR (CI)’s 0.81 (0.65, 1.01), 0.64 (0.53, 0.77), and 0.44 (0.21, 0.96), respectively). Women insured by Medicaid/Medicare, a proxy for low-income, were less likely to be screened postpartum than women who were privately insured (AOR (CI) 0.78 (0.68, 0.89)). National guidelines support universal depression screening of pregnant and postpartum women. The current study found opportunities for improvement in order to achieve universal screening and to deliver equitable care.

## Introduction

Prenatal and postpartum depression each have unique and significant risks for women and their infants. Prenatal depression (PND) is associated with spontaneous abortion, preterm delivery, pre-eclampsia, low birthweight, and neonatal growth retardation (Bennett et al. 2004; Bonari et al. 2004a; Davalos et al. 2012; Lusskin et al. 2007). Postpartum depression (PPD) is associated with maternal distress and may negatively impact maternal-infant bonding (Kingston et al. 2012; Lusskin et al. 2007). Infants of women experiencing PPD are at increased risk for cognitive and emotional developmental delays, and behavior problems as children (Kingston et al. 2012; Lusskin et al. 2007). Untreated prenatal and postpartum depression can result in poor adherence to medical care, substance abuse, suicide, and risk of infant mortality (Lusskin et al. 2007). Approximately 7–12% of women experience PND and 6–19% experience PPD (Bennett et al. 2004; Gavin et al. 2005; Gaynes et al. 2005). Rates of PND and PPD are associated with racial and socioeconomic disparities, with higher prevalence levels among low-income and African American women (Bennett et al. 2004; Goyal et al. 2010; Melville et al. 2010; Orr et al. 2006).

Despite its adverse consequences, depression is both underdiagnosed and undertreated in pregnant and postpartum women (Flynn et al. 2006; Kelly et al. 2001; Marcus et al. 2003), leading to more severe outcomes like hospitalizations, increased morbidity, and suicide (Bonari et al. 2004b). Women who experience one episode of perinatal depression are at increased risk of recurrence with subsequent pregnancies, highlighting the importance of identifying and treating depression early on in a woman’s reproductive life (Lusskin et al. 2007). Additionally, racial disparities may be exacerbated with higher rates of untreated depression among African American and Latina women (Kozhimannil et al. 2011). National attention has highlighted increasing rates of pregnancy-related mortality in the USA, characterized by significant racial disparities (Petersen et al. 2019b). However, surveillance methods for these deaths currently exclude deaths due to suicide or drug use, underrepresenting the contribution of mental health to maternal mortality (Mangla et al. 2019; Petersen et al. 2019a). One recent study estimated that 18% of all maternal deaths within 12 months of delivery may be due to suicide, or drug-related causes (Goldman-Mellor and Margerison 2019), highlighting the substantial need to improve identification and treatment of mental health conditions in pregnant and postpartum women.

Systematic screening has been demonstrated to increase the identification of depression (Avalos et al. 2016; Evins et al. 2000; Gaynes et al. 2005; O'Connor et al. 2016) and to be more effective at identifying depression than clinical assessment alone (Gaynes et al. 2005; Spitzer et al. 2000). Current guidelines from the United States Preventive Services Task Force (USPSTF) and the Council on Patient Safety in Women’s Health Care recommend screening women for depression at least once both during pregnancy and after delivery (Council on Patient Safety in Women’s Health Care 2016; Kendig et al. 2017; Siu et al. 2016). The American College of Obstetricians and Gynecologists (ACOG) recommends screening a minimum of once during the perinatal period and recommends a screen postpartum even if screening took place during pregnancy (ACOG Committee Opinion No. 757: Screening for Perinatal Depression 2018; Committee on Obstetric Practice 2015). All of these guidelines emphasize that screening alone is necessary but insufficient to address maternal depression, and providers must be prepared to initiate treatment as well as refer patients to mental health professionals when indicated (Committee on Obstetric Practice 2015; Council on Patient Safety in Women’s Health Care 2016; Kendig et al. 2017; Siu et al. 2016). Systems that incorporate depression screening with diagnostic assessment and clinical decision support are effective at improving clinical outcomes, patient acceptance of care, and increasing the capacity of obstetric providers to identify and treat depression (Miller et al. 2012; Siu et al. 2016).

Little information is available on the prevalence of depression screening of pregnant and postpartum patients as a component of routine obstetric care, and most research on screening frequency comes from provider surveys. However, self-reported screening behavior has been found to be inaccurate with most providers overestimating their screening prevalence (Kim et al. 2009). Few studies have examined screening rates in a health care setting beyond provider self-report (Avalos et al. 2016; Delatte et al. 2009; Kim et al. 2009). Additionally, while studies have documented disparities in diagnosis and treatment of depression (Kozhimannil et al. 2011; Simpson et al. 2007), less information is available on whether disparities exist in the delivery of depression screening initiatives.

The purpose of this study is to examine PND and PPD screening in the context of a large health system. Specific goals are to (1) identify depression screening prevalence among pregnant and postpartum patients and (2) identify patient or clinical factors associated with screening, including disparities in screening.

## Materials and methods

### Setting

This study took place at Allina Health, a large, non-profit health care system in Minnesota. Allina Health is the largest provider of obstetric services in Minnesota, with over 15,000 annual deliveries at 10 hospitals. Prenatal care at Allina Health clinics is provided by obstetrician-gynecologists (Ob-Gyns), family physicians (FPs), and certified nurse midwives (CNMs). The Mother Baby Clinical Service Line (MBCSL) is responsible for standardization of care delivery, clinical quality, and outcome metrics for inpatient and outpatient Ob-Gyn care and perinatology across the system.

Mental health services at Allina Health span the continuum of care, including outpatient psychiatry and psychotherapy, inpatient psychiatric units, partial hospital programs, and hospital and emergency department consultation-liaison services. The Mother Baby Mental Health Program (MBMHP) was started to focus on the long-term initiatives of increasing treatment options for pregnant and postpartum women, providing education and clinical decision support to obstetric providers, and implementing standardized care processes for common psychiatric conditions like depression in outpatient Ob-Gyn clinics. The MBMHP is staffed by a multidisciplinary team of psychiatrists, social workers/therapists, and nurses, and offers direct patient care, an electronic consultation service for perinatal providers, and education and training to obstetric providers and nurses in order to improve the care of women with perinatal psychiatric illness.

The MBCSL and MBMHP partnered to conduct this study to understand the current state of perinatal depression screening across the organization prior to the development of a standardized care process focused on increasing the identification and treatment of perinatal depression. This data includes women receiving prenatal care in 2015 and 2016; consequently, the majority of care occurred prior to the 2015 ACOG, 2016 USPSTF, and 2016 Council on Patient Safety in Women’s Health Care guidelines (Committee on Obstetric Practice 2015; Council on Patient Safety in Women’s Health Care 2016; Siu et al. 2016).

### Study sample and data collection

**Fig. 1:**
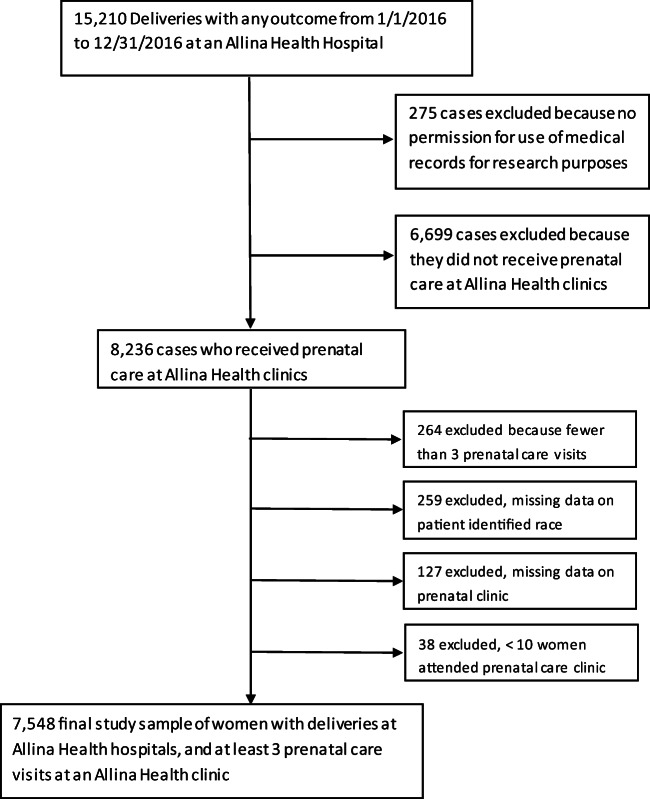
Study sample selection process

This retrospective cohort study used data from the electronic health record (EHR) to establish a cohort of women who received prenatal care in our system. Women included in the final data set: (1) delivered in 2016; (2) received at least three prenatal care visits at an Allina Health clinic; and (3) consent on file for use of their EHR data for research under the Minnesota Health Records Act. Women missing data on race or assigned to a clinic serving fewer than 10 prenatal care patients were excluded (Fig. 1). This study was approved by Allina Health’s Institutional Review Board.

### Measures

Maternal demographic measures included the following: age at the time of delivery, race (women selecting more than one race were coded as multi-racial), ethnicity (Hispanic/non-Hispanic), preferred language, marital status (categorized as single or married/partnered), and insurance type (categorized as private or Medicaid/Medicare). Race, ethnicity, and preferred language were all measured in accordance with the Meaningful Use Certification Criteria (Certification Companion Guide: Demographics 2019; Institute of Medicine 2009). Race and ethnicity are self-reported to clinic intake staff using a verbal script. These demographic measures were used to examine disparities as these are the key equity-related measures systematically collected in the electronic health record. Measures related to pregnancy included parity, singleton versus multiple gestation, provider type seen most for prenatal care, clinic of prenatal care (clinic the most common provider is affiliated with), number of prenatal care visits (3–9, 10–19, or 20+ to approximate low, average, and high utilization), pregnancy risk status (high or low), pregnancy outcome (live birth, miscarriage, fetal death, or abortion), gestational age at delivery (preterm for < 37 weeks or full-term for 37+ weeks), and birthweight (low for < 2500 g or normal for 2500+ g). Patients were classified as established or new patients based on whether they had any visits at an Allina Health clinic in the year proceeding pregnancy. The presence of depression or anxiety was identified based on ICD codes from the problem list or visit diagnosis for the year prior to pregnancy or the first prenatal care visit. Women were categorized as having a high-risk pregnancy if they experienced any of the following conditions coded during pregnancy: alcohol dependence/abuse not in remission, HIV, cocaine abuse, opioid abuse, diabetes (type 1, type 2, gestational), maternal bleeding or clotting disorders, multiple gestation, pre-eclampsia, poor fetal growth, morbidly adherent placenta (accreta, percreta, increta), or oligohydramnios. A pregnancy was considered low-risk in the absence of any high-risk conditions.

Outcome measures tracked included depression screening during pregnancy and the first 3 months postpartum. Screening was measured as completion of the Patient Health Questionnaire (PHQ)-9 (Kroenke et al. 2001; Spitzer et al. 1999) or PHQ-2 (Kroenke et al. 2003) which are embedded in the EHR. Screening is initiated by the PHQ-2, and the PHQ-9 is triggered if the responses to either question on the PHQ-2 are greater than 0. PHQ-2 and PHQ-9 screening dates were used to classify if screening occurred during pregnancy or the first 3 months postpartum. Screenings may have occurred at any clinic appointment type (e.g., office visit OB care, primary care, or a mental health visit). For patients with a preferred language other than English, interpreter services used at clinic sites for screening and other clinical communication include in person interpreters, bilingual staff, phone interpreters, and computer-based language services (used via a tablet). Gestational age of the initial prenatal PHQ-2 or PHQ-9 screen was calculated based on the patient’s expected delivery date and categorized by trimester.

Two clinic-level measures were created (by aggregating patient-level data) to describe the prenatal population served by each clinic during the study period: percent of patients insured through a government plan (Medicaid/Medicare) and percent of patients who were white, African American, Asian, and other non-white (Native American, multi-racial, Native Hawaiian, other Pacific Islander). Dichotomous indicators were created for each measure that coded clinics as serving a prenatal population above or below the grand mean for each variable, with dichotomous measures made for insurance and each of the four race measures.

### Analysis

Frequencies with chi-square statistics were used to examine associations of screening with maternal and clinical characteristics. Generalized linear mixed (GLM) models were used to determine the effect of maternal and clinical characteristics on depression screening for each time period. Women are nested in clinics; initial models were estimated (for each time period) to determine whether women who attend the same clinic are more similar on screening than women who attend different clinics (using the intraclass correlation coefficient (ICC)). The ICC of each model indicated the amount of variance in screening for both PND and PPD that could be explained by between-clinic differences. Significant factors from the maternal and clinical characteristics cross tabulations were incorporated as fixed effects into each model with clinic as a random effect to form a two-level model with random intercepts. Variables that were not significant in the adjusted models were removed to create the final two-level prenatal and postpartum mixed models. The final two-level postpartum mixed model was modified to include clinic-level predictors to further investigate observed disparities in PPD screening by clinic. All analyses were conducted using Stata version 15.

## Results

### Sample characteristics

Of the 8236 women who received prenatal care at Allina Health clinics, and delivered in 2016, we excluded 688 women who were missing data on key measures or who had fewer than 3 prenatal visits (Fig. 1). A comparison of those who were included in the final sample with those excluded found no significant differences with regard to parity, marital status, or age. The excluded group was more likely to have insurance through Medicaid/Medicare (43%) compared to the final sample (32%), *p* < 0.001, and had a higher proportion of women who spoke a language other than English (13.2%) compared to the final sample (6.2%), *p* < 0.001. The final sample included 7548 women receiving prenatal care at 35 clinics and delivering at 10 hospitals. Women in the sample were predominantly white, English speakers, married/partnered, and privately insured (Table 1). Most women (81%) received prenatal care from an Ob-Gyn provider, and about one-third (32.8%) were new patients at the start of prenatal care. A history of depression or anxiety was documented for 14.6% in the year prior to pregnancy or at the first prenatal care visit.

**Table 1:** Characteristics of study sample (*N* = 7548)

Study sample characteristics	Percent or mean ± SD
Age, mean **±** SD	29.7 **±** 5.34
Race	
African American	12.0%
American Indian/Alaska Native	0.6%
Native Hawaiian/other Pacific Islander	0.3%
Asian	7.4%
White	78.4%
Multi-racial	1.4%
Ethnicity	
Hispanic	5.3%
Preferred language	
English	93.9%
Somali	2.4%
Spanish	1.5%
Arabic	0.4%
Hmong	0.4%
Other	1.5%
Marital status	
Married/partnered	69.2%
Single	30.8%
Insurance	
Medicaid/Medicare	32.2%
Private	67.8%
Parity	
0	39.8%
1	32.9%
2	15.8%
3+	11.5%
Singleton or multiple gestation	
Singleton	95.7%
Multiple	4.3%
Prenatal care provider type (seen most often)	
Ob-Gyn doctor	81.0%
Family medicine doctor	11.8%
Nurse practitioner	4.5%
Certified nurse midwife	2.7%
Prenatal care visits, number of visits, mean **±** SD	11.9 **±** 3.36
3 to 9	19.9%
10 to 19	78.7%
20+	1.4%
Pregnancy risk status	
High-risk	21.6%
Low-risk	78.4%
Pregnancy outcome	
Live birth	99.2%
Miscarriage, fetal death, abortion	0.8%
Gestational age at delivery for live births, mean **±** SD	39.1 **±** 2.01
Preterm, < 37 weeks	7.6%
Full-term, 37+ weeks	92.4%
New or established patient	
Established	67.2%
New	32.8%
Prior or current depression/anxiety at start of prenatal care*	
No diagnosis of depression or anxiety	85.4%
Diagnosis of depression or anxiety	14.6%
Depression diagnosis only	3.9%
Anxiety diagnosis only	5.2%
Both depression and anxiety	5.5%

*Depression/anxiety history based on data from 1 year before first prenatal care visit or the first prenatal care visit. Diagnosis is based on problem list or visit diagnosis codes for depression or anxiety

To examine postpartum screening, the sample was limited to the 7059 (93.5%) women who returned within 3 months for either a postpartum visit (91.4%) or another type of face-to-face visit with either an Ob-Gyn, FP, or CNM. Women who did not return for postpartum care (6.5%, *n* = 489) were different from those who did (*n* = 7095). Specifically, 22.7% of American Indian, 11.5% of African American, and 8.8% of multi-racial women did not return for a postpartum visit compared to 5.5% of white women. Among women with public insurance, 17% did not return for postpartum care compared to < 1% of privately insured women.

### Prenatal screening prevalence, timing, and factors associated with screening

PND screening was conducted for 65.1% of the sample (Table 2), with 34.9% not screened at all, 52.0% screened once, and 13.3% screened two or more times during pregnancy. Among those who were screened (*n* = 4917), timing of initial screening was evenly spread across trimesters: 35.2% first trimester, 29.8% second trimester, and 35.0% third trimester.

**Table 2:** Examination of prenatal and postpartum depression screening by maternal, pregnancy, and provider characteristics

	Prenatal screening			Postpartum screening		
	Total prenatal sample	% screened during pregnancy	*p* value	Postpartum sample*	% screened postpartum	*p* value
Total	7548	65.1		7059	64.4	
Age						
24 and under	1324	64.7	0.380	1219	57.4	< 0.001
25–29 years	2321	64.4		2170	64.3	
30–34 years	2527	66.4		2391	66.8	
35 and over	1376	64.2		1279	67.0	
Race						
African American	908	62.4	0.394	802	48.9	< 0.001
American Indian/Alaska Native	44	61.4		34	47.1	
Asian	558	66.0		517	67.7	
Native Hawaiian/Pacific Islander	21	76.2		19	57.9	
White	5917	65.5		5594	66.6	
Multi-racial	102	62.8		93	55.9	
Ethnicity						
Hispanic	398	70.6	0.018	365	56.2	0.001
Not Hispanic	7150	64.8		6694	64.9	
Preferred language						
English	7081	65.4	0.055	6637	65.3	< 0.001
Language other than English	464	61.0		420	51.4	
Marital status						
Married/partnered	5216	64.6	0.136	4942	66.7	< 0.001
Single	2325	66.3		2110	59.3	
Insurance						
Medicaid/Medicare	2434	64.0	0.169	2177	56.4	< 0.001
Private	5114	65.6		4882	68.1	
Parity						
0	2973	66.2	0.092	2843	66.9	0.001
1+	4491	64.3		4140	62.9	
Number of prenatal care visits						
3 to 9	1501	57.1	< 0.001	1256	56.9	< 0.001
10 to 19	5937	66.9		5697	66.1	
20+	110	76.4		106	66.0	
Pregnancy risk status						
Low-risk	5916	65.1	0.929	5532	64.3	0.547
High-risk	1632	65.2		1527	65.1	
Single or multiple gestation						
Singleton	7224	65.5	0.001	6754	64.9	< 0.001
Multiple gestation	324	56.8		305	54.4	
Prenatal care provider type						
Ob-Gyn	6113	64.6	< 0.001	5702	67.2	< 0.001
Family medicine	890	78.4		847	51.6	
Nurse practitioner	340	45.9		311	55.6	
Certified nurse midwife	204	54.4		198	52.5	
Prenatal-only measures						
New or established patient						
Established	5072	66.0	0.021			
New patient	2476	63.3				
History of depression or anxiety at start of prenatal care						
History of depression or anxiety	1104	76.4	< 0.001			
No history of depression or anxiety	6444	63.2				
Postpartum-only measures						
Screened during pregnancy						
Yes				4642	64.1	0.438
No				2435	65.1	
Elevated symptoms during pregnancy						
Yes				521	69.5	0.007
No				4103	63.4	
Depression diagnosis during pregnancy						
Yes				672	68.5	0.022
No				6387	64.0	
Pregnancy outcome						
Miscarriage/abortion				15	73.3	0.507
Fetal or infant death				40	57.5	
Live birth discharged alive				7004	64.5	
Gestation at delivery (singletons only)						
Preterm, < 37 weeks gestation				476	64.3	0.768
Full-term, 37+ weeks gestation				6266	65.0	
Birthweight (singletons only)						
Low birthweight, < 2500 g				415	66.0	0.619
Birthweight of 2500+ g				6339	64.8	

Note percentages shown are within rows

*The postpartum sample includes women from the prenatal sample who returned within 0–3 months postpartum for a postpartum visit or other face-to-face visit with one of the provider types included in our definition of a prenatal care visit

In unadjusted cross-tabulations, there were substantial differences in prenatal screening prevalence by prenatal care provider type, with patients of family medicine doctors most likely to get screened (78.4%), followed by Ob-Gyn patients (64.6%), with lower rates among patients primarily seen by certified nurse midwives (54.4%) or nurse practitioners (54.5%). Substantial differences were also seen by number of prenatal care visits (higher screening with more visits), history of depression or anxiety (increased screening for those with a history), and by specific clinic (Table 2). There were small differences (< 5 percentage points) by ethnicity and between existing and new patients. There were no differences in PND screening prevalence when examined by patient age, race, preferred language, marital status, insurance type, parity, pregnancy risk status, and singleton/multiple gestation.

PND screening prevalence ranged widely by clinic, from a low of 34.7% to a high of 100% of pregnant patients across the 35 clinics. Related, the intraclass correlation indicates that almost a quarter of the variance in whether a woman was prenatally screened can be explained by the clinic she attended for prenatal care (ICC = 0.23). After adjusting for these clinic differences in the GLM regression model, preferred language, number of prenatal care visits, and history of depression or anxiety were significantly associated with PND screening (Table 3). In adjusted models, women who spoke a language other than English were less likely to be screened prenatally (AOR 0.74). Women with more prenatal care visits were more likely to get screened, and women with a documented history of depression or anxiety were twice as likely to get screened (AOR 2.18) as women without a documented history of these conditions.

**Table 3:** Generalized linear mixed model of factors associated with prenatal depression screening, grouped by clinic (*n* = 7545)

	OR	CI	*p* value
Preferred language			
English	Ref.		
Language other than English	0.74	[0.59, 0.92]	0.007
Number of prenatal care visits, categories			
3 to 9	Ref.		
10 to 19	1.63	[1.43, 1.85]	< 0.001
20+	2.26	[1.38, 3.68]	0.001
History of depression or anxiety at start of prenatal care			
None documented	Ref.		
Depression or anxiety diagnosis documented	2.18	[1.85, 2.56]	< 0.001

### Postpartum screening prevalence and factors associated with screening

As noted previously, 6.5% of those with prenatal care did not return for a visit within 3 months postpartum. Of the women who returned for postpartum care within 3 months, 64.4% were screened for depression (Table 2).

Variations in screening practices that were not present during pregnancy were identified postpartum. Disparities related to age, race, ethnicity, language, marital status, insurance, and parity as well as provider type and prenatal mental health status emerged (Table 2). Specifically, women were less likely to get screened if they were young (< 24 years), African American, American Indian, multi-racial, Hispanic, single, insured by Medicaid/Medicare, or spoke a language other than English. Screening rates also differed by provider type, but with a different pattern than seen prenatally. Contrary to prenatal screening patterns, women who received care from an Ob-Gyn were most likely to get screened postpartum (71.2%) compared to patients served by other provider types (58–60%). We found no difference in screening by birth outcome, gestational age at delivery, or birthweight. Similar to results seen in pregnancy screening, women who had an identified history of depression (specifically elevated screening results or a depression diagnosis in pregnancy) were more likely to get screened postpartum (76%) compared to those without a positive screen or diagnosis (67–68%).

Postpartum screening varied widely by clinic, ranging from 24.8 to 95.6% among the 35 clinics. As with the prenatal screening models, individual clinic was the most important factor predicting the likelihood of postpartum screening, accounting for 30% of the variability in postpartum screening prevalence. Disparities were still present in the final model after adjusting for clinic using a random intercept for clinic (Table 4). In the final model, women who were African American, Asian, and otherwise non-white (Native American, multi-racial, Alaska Native) were less likely to be screened than white women (AOR’s 0.64, 0.81, and 0.44, respectively). Women insured by Medicaid/Medicare, a proxy for low-income, were less likely to be screened than women who were privately insured (AOR 0.78). Women with a parity of 1+ were less likely to get screened (AOR 0.77) than nulliparous women. Screening for depression during pregnancy was inversely associated with postpartum screening (AOR 0.83). Women with a depression diagnosis during pregnancy were more likely to get screened (AOR 1.56) than women with no prenatal diagnosis of depression. Additionally, women with 10–19 prenatal care visits were more likely to get screened postpartum (AOR 1.27) than women with fewer visits.

**Table 4:** Generalized linear mixed model of factors associated with postpartum depression screening among women who had a visit within 3 months postpartum (*n* = 6872)

	OR	CI	*p* value
Age			
24 and under	Ref.		
25–29 years	1.24	[1.04, 1.48]	0.016
30–34 years	1.34	[1.12, 1.61]	0.002
35 and over	1.43	[1.16, 1.76]	0.001
Race			
White	Ref.		
Asian	0.81	[0.65, 1.01]	0.064
African American	0.64	[0.53, 0.77]	< 0.001
Native American, Hawaiian, Alaska Native, multi-racial	0.44	[0.21, 0.96]	0.040
Insurance			
Private	Ref.		
Medicaid/Medicare	0.78	[0.68, 0.89]	< 0.001
Parity			
0	Ref.		
1+	0.77	[0.68, 0.87]	< 0.001
Screened during pregnancy			
No	Ref.		
Yes	0.83	[0.73, 0.95]	0.008
Depression diagnosis during pregnancy			
No	Ref.		
Yes	1.56	[1.27, 1.91]	< 0.001
Number of prenatal care visits			
3 to 9	Ref.		
10 to 19	1.27	[1.09, 1.48]	0.002
20+	1.15	[0.7, 1.87]	0.586

Sample size reduced due to missing data

We did two sets of exploratory analyses to further examine the role of clinic in depression screening. First, we incorporated two clinic-level predictors to attempt to explain the clinic-level screening differences indicated by the nonzero ICC. We added binary indicators for insurance (above or below mean proportion of government insurance) and each of the 4 race groups (above or below mean proportion of African American, Asian, other non-white, and white). The only one of these variables that was associated with postnatal screening was clinics with a higher than average Asian population; clinics serving a larger than average proportion of Asian women had over twice the odds of postnatal screening (AOR = 2.38, 1.11–5.14, *p* = 0.027). Given that Asian women in general were less likely to get screened, it is possible that this clinic-level measure is a proxy for other clinic-level factors related to screening (in addition to race of patients served).

Second, we estimated a set of models examining whether the effects of patient race and insurance type were differentially associated with postnatal screening across clinics (i.e., cross-level interactions between clinic and these variables using random slope models). The purpose of these models was to assess if disparities identified in screening vary between clinics. We found no evidence that the association between postnatal depression screening and patient insurance and race varied across clinics, indicating the disparities in screening were occurring similarly across clinic sites.

## Discussion

Our study found 65.1% of prenatal and 64.4% of returning postpartum patients were screened for depression. These prevalence levels represent screening practices just prior to publication of current guidelines recommending universal screening during and after pregnancy (ACOG Committee Opinion No. 757: Screening for Perinatal Depression 2018; Committee on Obstetric Practice 2015; Siu et al. 2016). Clinic site accounted for the majority of the variability in depression screening rates. Our study also identified significant racial and socioeconomic disparities in postpartum screening.

We found few other studies documenting screening rates across an entire health system. One study assessed the screening rates for 22 providers and found a range of screening by provider from 5 to 95% of patients with an average of 52% of prenatal patients screened. Notably, this study compared actual screening rates to self-reported estimates and found 95% of providers overestimated their screening rates, generally by twofold the actual rate (Kim et al. 2009). Another study documented an increase from < 1% of perinatal patients getting screened prior to the implementation of a system-wide universal screening program to 98% after full implementation (Avalos et al. 2016).

Little research is available identifying factors associated with screening prevalence. Similar to our findings, a few studies identified clinic as a major predictor of screening behavior (Fedock and Alvarez 2018; Kim et al. 2009). A national obstetric provider survey found the most influential factor associated with self-reported universal screening was working in a clinic where depression screening was an identified clinic priority (Fedock and Alvarez 2018). Similarly, among providers with high screening rates, factors associated with increased screening were identified as an established prompt inside the EHR, inclusion of screening as part of established procedures at the clinic, and having a nurse champion invested in ensuring the screening and follow-up is standardized (Kim et al. 2009). In our study, provider type was associated with both prenatal and postpartum screening, with highest prevalence of prenatal screening occurring among patients of family physicians and the highest prevalence of postpartum screening among patients of Ob-Gyns. The prenatal pattern is likely related to a system-wide primary care initiative to screen all adults for depression annually. Women receiving prenatal care from a family physician may have been more likely to get screened as a result of this policy independent of pregnancy status.

A particularly troubling finding in our data was the racial and socioeconomic disparities in postpartum screening practices. We found no other studies that examined patient-level demographic or racial disparities in screening prevalence. Given that these disparities were not present prenatally, the explanation is not likely due exclusively to provider bias. Clinic workflow related to PPD screening and variations in patient population across clinics are both potential explanations. However, we were not able to explain these disparities by adjustment for variation in clinic or clinic-level patient population measures. Rather these disparities appeared to exist within clinics, regardless of the proportion of clinic patients that were low-income. The disparities found in this study are of particular concern because the same women who are at greatest risk for depression (i.e., low-income or African American) (Bennett et al. 2004; Goyal et al. 2010; Melville et al. 2010; Orr et al. 2006) as well as disparities in treatment for depression (Kozhimannil et al. 2011) were also those least likely to be screened postpartum in our study. Disparities in screening, such as those found in our study, likely contribute to disparities in detection and treatment. Gaps in screening are also concerning in light of the potential contributions of mental health (Goldman-Mellor and Margerison 2019) to increasing maternal mortality and associated disparities (Petersen et al. 2019b). Additionally, our study found that 6.5% of women did not return for a visit within 3 months postpartum, leaving no opportunity for postpartum screening. As noted in the “Results” section, these women also included higher proportions of demographic groups most at risk for depression. Understanding the differences in who returns for a postpartum visit offers insight into the value of screening during pregnancy as an opportunity to screen all women as some may not return. These disparities also highlight the need for strategies to increase postpartum visit attendance for high-risk populations and integration of screening into pediatric visits (Earls et al. 2019).

While we were not able to explain the cause of the discrepancy in screening rates identified in our sample of women postpartum, our findings identify a need to increase both prenatal and postpartum screening practices to align with current recommendations. Findings from this study helped make the case for prioritization of activities to promote screening across our health system. The primary strategy implemented was the addition of depression screening rates as a quality metric on a system-level scorecard, reviewed regularly at the organization’s OB Quality Committee attended by clinic managers and providers. Additionally, the MBMHP team developed guidance for providers in how to best address screening results, initiate treatment, and refer for mental health care when indicated. The team also provided education, training, and clinical decision support for outpatient providers and nurses in order to improve the identification of and treatment of women with perinatal mental health conditions.

Research on health care quality has documented across many health care fields and specialties, a significant time gap between release of clinical guidelines and integration into standard care (Institute of Medicine and Committe on Standards for Developing Trustworthly Clinical Practice Guidelines 2011; McGlynn et al. 2003; Morris et al. 2011). Integration requires a process of education about new guidelines, modifying clinical practice and provider behavior to adopt recommendations, and providing the infrastructure to support these activities (Institute of Medicine and Committe on Standards for Developing Trustworthly Clinical Practice Guidelines 2011). Within our own organization, limited resources, organizational restructuring, and staff turnover created barriers to implementing and spreading best practices for perinatal depression screening across the system. One recommendation for improving provider adoption of clinical guidelines includes the use of audit and feedback reporting, which aligns with our scorecard strategy (Institute of Medicine and Committe on Standards for Developing Trustworthly Clinical Practice Guidelines 2011). Given the expected time lag from the guideline release to adoption, a future step in this line of research will be to examine the trends within the health system to assess changes in screening prevalence and related disparities.

One strength of the study is the inclusion of an entire health system population, allowing the identification of opportunities to address gaps in care with programmatic initiatives. Additionally, the incorporation of the screening tool into the EHR enables accurate measurement of screening rates that is not found in prior studies relying on provider or patient self-report. Our study was limited to what data was available in the EHR, which in this case was not sufficient to explain the disparities identified. Additionally, our population may not be generalizable to other communities based on differences in demographics and postpartum visit attendance.

## Conclusion

National guidelines support universal depression screening of pregnant and postpartum women. This study identified opportunities for improvement to achieve universal screening and address equitable care.
